# Decision Support Model for Introduction of Gamification Solution Using AHP

**DOI:** 10.1155/2014/714239

**Published:** 2014-04-23

**Authors:** Sangkyun Kim

**Affiliations:** Kangwon National University, Chuncheonsi, Gangwondo 200-701, Republic of Korea

## Abstract

Gamification means the use of various elements of game design in nongame contexts including workplace collaboration, marketing, education, military, and medical services. Gamification is effective for both improving workplace productivity and motivating employees. However, introduction of gamification is not easy because the planning and implementation processes of gamification are very complicated and it needs interdisciplinary knowledge such as information systems, organization behavior, and human psychology. Providing a systematic decision making method for gamification process is the purpose of this paper. This paper suggests the decision criteria for selection of gamification platform to support a systematic decision making process for managements. The criteria are derived from previous works on gamification, introduction of information systems, and analytic hierarchy process. The weights of decision criteria are calculated through a survey by the professionals on game, information systems, and business administration. The analytic hierarchy process is used to derive the weights. The decision criteria and weights provided in this paper could support the managements to make a systematic decision for selection of gamification platform.

## 1. Introduction

Motivation of organizational members is one of the most important things and is not an easy problem to solve in corporate and education environments [1]. The study on gamified class showed that the gaming approach is both more effective in improving students' knowledge and more motivational than the nongaming approach [2]. The work of [3] showed that gamification is effective in work collaboration. However, it is a dawn of research on gamification and hard to find the detailed methodologies or works which show how the gamified environments could be designed and developed.

To improve the organizational outputs of the gamified environments and to motivate the organizational members with the gamified environments effectively, the gamified environments should be designed and developed according to the interdisciplinary approaches including psychology, computer science, pedagogy, management science, economics, esthetics, demography, statistics, and industrial engineering because the basis of the gamified environments is a game and those studies are closely related with a game. Managements might easily face with the problem when they select and introduce the gamification platform because the gamification platform has convergent characteristics of interdisciplinary areas described above. Providing a systematic decision making method for gamification process is the purpose of this paper. This paper suggests the decision criteria and weights for the selection of gamification platform to support a systematic decision making process for managements.

The following parts of this paper are organized in three parts. Firstly, previous works are reviewed including the recent approach of gamification, methodologies for selection and introduction of information systems, and analytic hierarchy process (AHP). Secondly, the decision criteria, weights, and a case study are provided. Finally, the implication of this study and further research issues are summarized in the Conclusion section.

## 2. Related Work

Previous works on the following topics are summarized in this section. Firstly, the definition and trend of gamification research and previous works on gamification supporting platforms are summarized. Secondly, the methodologies for selection and introduction of information systems are reviewed because the gamification supporting platform is a kind of the information systems. Thirdly, the concept and previous works on AHP are provided.

### 2.1. Gamification and Supporting Platforms

Gamification is defined as the use of various elements which could be used in game design in nongame contexts including workplace collaboration, marketing, education, military, and medical services [4]. The recent works on gamification are summarized in Table 1.

The work of [5] defined the gamification platform as “It comes complete with reward features for points, levels, badges, virtual goods, Facebook credits, and coupons. There are installable widgets for notifications, progress, avatars, profiles, leaderboards, social sharing. There are published APIs for deep integration, back-end admin consoles for set up, and full reporting and metrics.” There are many kinds of platforms for gamification. The list of some leading platforms includes Gamify, Badgeville, Bunchball, Big Door Media, CrowdTwist, Cynergy, SpectrumDNA, Reputely, iActionable, Scvngr, Manumatix, and Leapfrog Builders.

### 2.2. Methodologies for Selection and Introduction of Information Systems

METHOD/1 supports the introduction of enterprise information systems and breaks down each phase of introduction process into smaller steps named segments and tasks. A series of manuals of METHOD/1 provides these steps in detail [6, 7].

ASAP is SAP's rapid implementation supporting tool designed to streamline and standardize the implementation of SAP products. ASAP aims to optimize time, quality, and efficient use of resources. ASAP supports the entire team which includes internal team members from the customer company and external consultants such as project manager, business process consultants, and the technical staffs.

The work of [8] proposed integrated methodology framework which is composed of patterns, scenarios, road map, components, and repository. The components offer detailed functional tools needed in the implementation path, which includes the support system for solution introduction and evaluation.

The work of [9] provides methodology which consists of process and criteria to support selection activities of the information security systems. It presents the rating approach for prioritizing security systems and the hierarchical structure of process and criteria.

The work of [10] summarized quality attributes of software products. They found that there are different schools/opinions/traditions concerning the properties of critical systems and the best methods to develop them are performance (from the tradition of hard real-time systems and capacity planning), dependability (from the tradition of ultrareliable, fault-tolerant systems), security (from the traditions of the government, banking, and academic communities), and safety (from the tradition of hazard analysis and system safety engineering).

ISO/IEC 9126 provides an international standard for the evaluation of software quality. ISO/IEC 9126 aims to solve the problems of human biases that could cause a negative impact on the selection and introduction of software. The human biases include unclear goal of the project, changing priorities after the kickoff of a project. To solve these problems, ISO/IEC 9126 suggests common goals of software selection and introduction projects which are as follows [11].

- Functionality: a set of attributes which provide a set of functions and their specified properties. These attributes provide suitability, accuracy, interoperability, security, and functionality compliance.
- Reliability: a set of attributes which guarantee the performance level under stated conditions for a stated period of time. These attributes provide maturity, fault tolerance, recoverability, and reliability compliance.
- Usability: a set of attributes which make easy to use, and the individual assessment of use by a set of users. These attributes provide understandability, learnability, operability, attractiveness, and usability compliance.
- Efficiency: a set of attributes which guarantee the effective balance between inputs and outputs of the system. The inputs mean the amount of resources used for the system. The outputs mean the performance level of the system. The attributes provide time behavior, resource utilization, and efficiency compliance.
- Maintainability: a set of attributes which mean easiness for specified modifications of the system. These attributes provide analyzability, changeability, stability, testability, and maintainability compliance.
- Portability: a set of attributes which support the system to be transferred from one environment to another. These attributes provide adaptability, installability, coexistence, replaceability, and portability compliance.

### 2.3. AHP

The AHP is a structured technique which supports a complex situation of decision making. It was proposed by Saaty in the 1970s based on mathematics and has been widely studied and used since then. It can be used in group decision making situation and has been used in various fields such as education, industry, and government.

Using the AHP, the decision problem is decomposed and structured into a hierarchy of easily understandable subproblems. One of the most important things is that each subproblem should guarantee independency. Each subproblem might be tangible or intangible aspect of the decision problem. After the building of the structured hierarchy, the decision makers judge pairwise comparison for every element of subproblems. The pairwise comparison is the process which judges the relative impact or importance of each element. In the pairwise comparison process, the decision makers can use concrete data about the elements or use their intuitive and professional judgments about the elements. Using of the human judgments is the essence of the AHP.  Table 2 summarizes previous works on AHP.

## 3. Decision Supporting Model Using AHP

This section provides the decision criteria and weights for the selection of gamification platform. The decision criteria are derived from previous works on gamification and information systems.

The weights of decision criteria are calculated through a survey by the professionals using AHP. Also, a case study is provided to show a functionality and practical value of the decision criteria and weights.

### 3.1. Decision Criteria for Gamification Platform

This paper takes [8, 9] to suggest the first and second level of criteria. [3, 10–17] are used to derive third level of criteria. The decision criteria for the selection of gamification platform are shown in Table 3.

### 3.2. Weights of Decision Criteria for Gamification Platform

Judgments were elicited from the eight professionals on game, information systems, and business administration.

**Figure 1:**
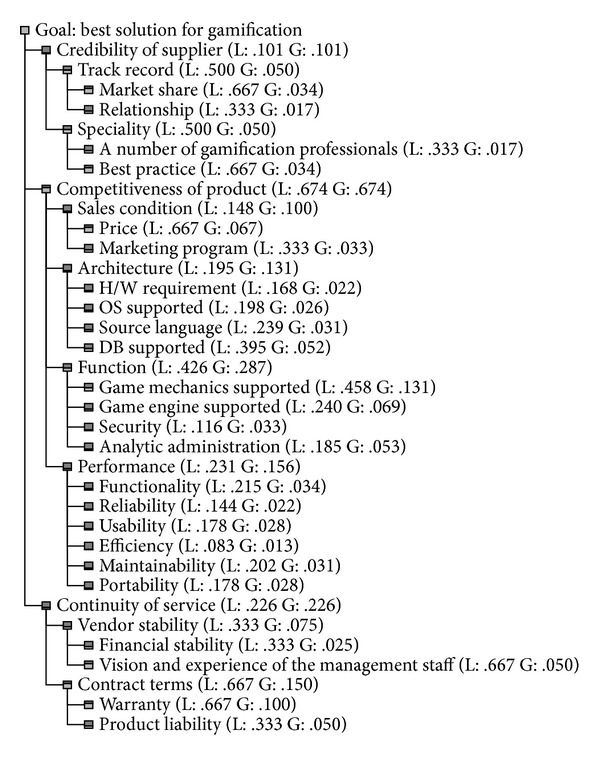
Weights of decision criteria for gamification platform.

Expert Choice was used to rate the priorities among criteria. For example, the competitiveness of product was the most important criteria in level 2. After inputting the criteria and their importance into Expert Choice, the priorities from each set of judgments were found and recorded as shown in Figure 1.

The decision model classifies the goal, decision criteria, and variables into four major levels. The highest level of the hierarchy is the overall goal, to select the best gamification platform. Level 2, level 3, and level 4 represent the criteria in selecting the gamification platform. The overall consistency of the input judgments at all levels is within the acceptable ratio of 0.1, as recommended by Saaty et al. [18].

## 4. A Case Study

### 4.1. Background

In this case study, AHP and the proposed selection model for gamification platform were applied to a particular project in which X Company located in South Korea wanted to select gamification platform. There was no relationship in corporate governance structure between gamification platform vendors and X Company, so vendors and products were treated as independent. Three gamification platforms were prepared for decision alternatives. In this paper, the alternatives are called platform A, platform B, and platform C.

### 4.2. Comparative Judgments on Three Gamification Platforms

Five staffs participated to compare each product using Expert Choice software.  Table 4 shows the normalized priority weights of the gamification platforms.

**Figure 2:**
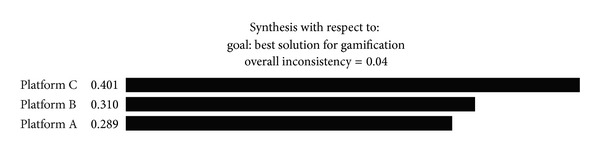
Synthesis result of the selection problem (exported from Expert Choice).

The overall priority of the gamification platform alternatives is calculated by multiplying its global priority with the corresponding weight along the hierarchy. Synthesizing all the elements using Expert Choice, the results are shown in Figure 2. It shows that gamification platform C scored the highest in the result, followed by platform B and platform A.

### 4.3. Sensitivity Analysis

**Figure 3:**
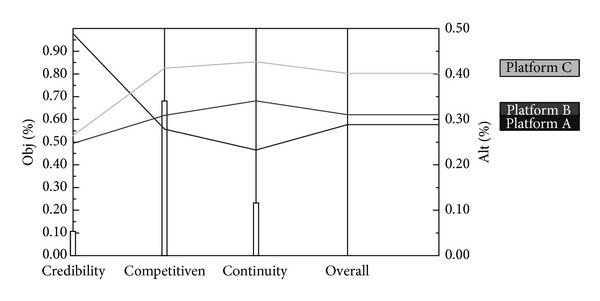
Sensitivity analysis (exported from Expert Choice).

Sensitivity analysis attempts to check the impact of change in the input data or parameters of the proposed gamification platform. Relatively small changes in the hierarchy or judgment may lead to a different outcome. Using Expert Choice, the sensitivity of the outcome can be tested.  Figure 3 shows a sensitivity analysis of the alternative priorities with respect to changes in the relative weights of the criteria.

### 4.4. Validation

The goal of the validation was to ensure that the results derived from this model were reasonable. That is to say, to examine if the gamification expert's knowhow could be substituted with the proposed decision criteria and weighted priorities is the goal of this validation.

Three gamification experts, apart from five staffs who participated in comparative judgments on three gamification platforms, helped to validate the proposed model. The judgments of three gamification experts with their own gamification knowhow and the result derived from this model as described in Section 4.2 were compared to assess the functionality of this model. Three gamification experts were not informed of which platform had been selected using this model. Background information on X Company and whitepapers on three gamification platforms were provided to gamification experts. After reviewing this information, three gamification experts chose the best platform and the worst platform for X Company. They chose platform C as the best and platform A as the worst. The gamification experts' choice based on their knowhow was matched with the result described in Section 4.2.

## 5. Conclusion

This paper provides the decision criteria and weights for the selection of gamification platform. The decision criteria are derived from previous works on gamification and information systems. The weights of decision criteria are calculated through a survey by the professionals using AHP. Also, a case study that X Company used the decision criteria and weights for the selection of gamification platform in is provided to show a functionality and practical value of this paper.

The implications of this paper are summarized as follows.

- As described at the introduction part of this paper, it is only a short time since gamification approaches began, so the decision criteria and weights provided in this paper could support the selection of gamification platform.
- The decision criteria on gamification platform would support the managements to understand what they should consider for successful gamification.

Limitation and further research issues are summarized as follows.

- It lacks providing sufficient pool of survey respondents for pairwise comparison, so a number of survey respondents should be increased to improve the reliability of the weights of decision criteria for gamification platform.
- A case study which validates the functionality of the decision criteria for gamification platform is provided. However, the validation is not suffonsified because it only provides a single case.
- Decision criteria for gamification platform should be enriched and revised through the in-depth and interdisciplinary reviews on human behavior, theory of organizational structure, theory of organizational behavior, content theory, process theory, game design, information systems, and so on.

## Figures and Tables

**Table 1:** Previous works on gamification.

Previous works	Key characteristics
[12]	It classifies the gamification approaches into the following: focusing on the technological aspects of computer games, focusing on the behavior evoked by computer games, and focusing on the design of computer games. It provides some case studies which show the process and benefits of gamification

[13]	It describes the characteristics of generation Y and the key elements of games that deserve a place in the enterprise including performance, achievement, and social interaction

[14]	It shows the interaction matrix of basic human desires and game mechanics including points, levels, challenges, virtual goods, leaderboards, and gifting. It provides the recent cases of gamification such as the frequent flyer programs, Foursquare, and Nike Plus

[15]	It describes the concept, benefits, key elements and mechanics of game design, and various cases of gamification

[3]	It shows the patterns of user activity in an enterprise social network service after the removal of game elements. It proved that the removal of game elements reduced overall participation within the SNS

[16]	It provides an experiment in computer science class which provides more frequent commits using a social software application. This study shows that the game elements are effective to motivate engineering students

[17]	It provides examples of social games which show behavioral economic biases related to the loss aversion tendency which is one of the key factors of behavioral economics and prospect theory

[19]	It provides the classification of engineering students based on the Bartle's game player types using the online survey which consists of 24 questionnaires

**Table 2:** Previous works on AHOP.

Previous works	Key characteristics
[18, 20]	It describes the definition, calculation procedures, and application areas of AHP method

[21]	It provides a decision making model for selection of automobile using AHP

[22]	It provides the decision criteria and decision model, which are based on AHP, for introduction of expert system which could be used in education environments

[23]	It shows a decision model which supports an introduction of multimedia authoring tool for multiple decision makers

**Table 3:** Decision criteria for gamification platform.

1st level criteria	2nd level criteria	3rd level criteria
Credibility of supplier	Track record	Market share Relationship
	Speciality	A number of gamification professionals Best practice

Competitiveness of product	Sales condition	Price Marketing program
	Architecture	Hardware requirement OS supported Source language DB supported
	Function	Game mechanics supported Game engine supported Security Analytic administration
	Performance	Functionality Reliability Usability Efficiency Maintainability Portability

Continuity of service	Vendor stability	Financial stability Vision and experience of the management staff
	Contract terms	Warranty Product liability

**Table 4:** Normalized priority weights of three gamification platforms.

Decision criteria and weights			Platforms' priority weights		
Level 1	Level 2	Level 3	Platform A	Platform B	Platform C
Credibility of supplier (L: 0.101 G: 0.101)	Track record (L: 0.500 G: 0.050)	Market share (L: 0.667 G: 0.034)	0.019	0.01	0.006
		Relationship (L: 0.333 G: 0.017)	0.002	0.003	0.009
	Speciality (L: 0.500 G: 0.050)	A number of gamification professionals (L: 0.333 G: 0.017)	0.009	0.004	0.003
		Best practice (L: 0.667 G: 0.034)	0.019	0.007	0.008

Competitiveness of product (L: 0.674 G: 0.674)	Sales condition (L: 0.148 G: 0.100)	Price (L: 0.667 G: 0.067)	0.011	0.02	0.037
		Marketing program (L: 0.333 G: 0.033)	0.003	0.006	0.019
	Architecture (L: 0.195 G: 0.131)	H/W requirement (L: 0.168 G: 0.022)	0.006	0.012	0.012
		OS supported (L: 0.198 G: 0.026)	0.014	0.006	0.005
		Source language (L: 0.239 G: 0.031)	0.004	0.009	0.017
		DB supported (L: 0.395 G: 0.052)	0.029	0.012	0.008
	Function (L: 0.426 G: 0.287)	Game mechanics supported (L: 0.458 G: 0.131)	0.016	0.073	0.042
		Game engine supported (L: 0.240 G: 0.069)	0.007	0.011	0.038
		Security (L: 0.116 G: 0.033)	0.002	0.006	0.019
		Analytic administration (L: 0.185 G: 0.053)	0.026	0.011	0.029
	Performance (L: 0.231 G: 0.156)	Functionality (L: 0.215 G: 0.034)	0.019	0.009	0.009
		Reliability (L: 0.144 G: 0.022)	0.012	0.004	0.002
		Usability (L: 0.178 G: 0.028)	0.015	0.008	0.008
		Efficiency (L: 0.083 G: 0.013)	0.002	0.004	0.007
		Maintainability (L: 0.202 G: 0.031)	0.003	0.006	0.017
		Portability (L: 0.178 G: 0.028)	0.015	0.006	0.003

Continuity of service (L: 0.226 G: 0.226)	Vendor stability (L: 0.333 G: 0.075)	Financial stability (L: 0.333 G: 0.025)	0.014	0.007	0.007
		Vision and experience of the management staff (L: 0.667 G: 0.050)	0.024	0.028	0.011
	Contract terms (L: 0.667 G: 0.150)	Warranty (L: 0.667 G: 0.100)	0.008	0.031	0.056
		Product liability (L: 0.333 G: 0.050)	0.008	0.015	0.028
